# Foundations of information technology based on Bunge’s systemist philosophy of reality

**DOI:** 10.1007/s10270-021-00862-5

**Published:** 2021-01-18

**Authors:** Roman Lukyanenko, Veda C. Storey, Oscar Pastor

**Affiliations:** 1 HEC Montreal, Montreal, Canada; 2 Georgia State University, Atlanta, Georgia USA; 3 Universitat Politècnica de València, Valencia, Spain

**Keywords:** Ontology, Upper-level ontology, General ontology, Mario Bunge, Bunge–Wand–Weber ontology, Bunge’s Systemist Ontology, Conceptual modeling, Software engineering, Database design, IT development, IT design, Real-world domains, Reality, Philosophy

## Abstract

General ontology is a prominent theoretical foundation for information technology analysis, design, and development. Ontology is a branch of philosophy which studies what exists in reality. A widely used ontology in information systems, especially for conceptual modeling, is the BWW (Bunge–Wand–Weber), which is based on ideas of the philosopher and physicist Mario Bunge, as synthesized by Wand and Weber. The ontology was founded on an early subset of Bunge’s philosophy; however, many of Bunge’s ideas have evolved since then. An important question, therefore, is: do the more recent ideas expressed by Bunge call for a new ontology? In this paper, we conduct an analysis of Bunge’s earlier and more recent works to address this question. We present a new ontology based on Bunge’s later and broader works, which we refer to as *Bunge’s Systemist Ontology (BSO)*. We then compare BSO to the constructs of BWW. The comparison reveals both considerable overlap between BSO and BWW, as well as substantial differences. From this comparison and the initial exposition of BSO, we provide suggestions for further ontology studies and identify research questions that could provide a fruitful agenda for future scholarship in conceptual modeling and other areas of information technology.

## Introduction

Human society is relentlessly increasing its reliance on information technology (IT). This reliance will only grow stronger as a result of the COVID-19 pandemic, providing a new impetus to move even more human activities online [116, 120]. The human world is becoming digital, which is happening especially rapidly since the last decade and a half [49, 88, 122]. It is thus particularly concerning that the IT projects that support this digitalization frequently fail [59, 81]. IT usability is often low [44, 100]; digital data continues to be of poor quality [4, 39]. These problems have a common characteristic in that they either directly or indirectly deal with how IT shapes and represents real-world domains.

It is critical to build IT based on solid theoretical and methodological foundations [55, 62, 117]. However, IT development often continues to be conducted in an ad hoc manner, with the outcomes heavily dependent on the skills and training of developers [3, 42, 84]. At its core, information technologies manipulate symbols making it further important to ensure that the relationship between the symbols upon which IT is based, is anchored appropriately in their real-life referents [117]. For example, the physical inventory of cars at a dealership may be represented symbolically using binary patterns stored on a computer hard drive and managed and organized by a database management system. The database, in turn, can be accessible to prospective buyers over the internet via a web interface. In order for the prospective customers of the dealership to gain an accurate knowledge of what cars are actually available, it is essential to ensure that correct patterns of bits and bytes are properly governed by the database management system. The patterns, in turn, must be correctly designed based on the accurate model of the car dealership domain. Hence, the goal of building better IT involves the investigation of the relationship between what is being stored and manipulated in a computer and its real-world referents.

Historically, one of the most prolific and effective foundations for IT analysis, design and development has been *ontology*. Ontology is a branch of philosophy that studies what exists in reality, as well as what reality is [53, 56]. In this research, we focus on a *general ontology*, also known as a foundational or upper-level ontology. A general ontology can provide IT development with theoretically grounded, consistent, formalized and rigorous meaning for the basic notions of what exists in reality.^1^

Due to their potential to put IT development on stronger methodological foundations, ontological studies are widely embraced by the IT community. Applications are especially prolific in research on *semantic web* [9, 32], which aims to move beyond syntactic matches to deeper interoperability, and on *conceptual data and process modeling* which develops representations of application domains and user requirements [77, 80, 88]. Ontologies have also been used in knowledge management, artificial intelligence, interface design, database schema integration, analysis of software performance, information quality, and other applications [47, 54, 56, 85, 90, 91, 101, 104, 109, 119]. Empirical benefits of adopting a specific domain ontology for conceptual modeling or to improve data quality have been documented [8, 12, 35, 37, 72, 89, 102, 104].

Various general ontologies have been used for IT analysis, design and development. Prominent examples include Unified Foundational Ontology (UFO) [57], social ontology of Searle [76], General Formal Ontology [63], DOLCE [50], Phenomenological Foundational Ontology (PFO) [67], ResearchCYC [38], and others (for more discussion, see, e.g., [56]).

A major ontology for conceptual modeling and other IT applications is the Bunge–Wand–Weber (BWW), based on works of the philosopher and physicist Mario Bunge (1919–2020), and synthesized and applied by Wand and Weber and colleagues [108, 110, 114]. The BWW has been applied in theoretical, empirical and design research across a wide range of disciplines [33, 115]. It has also provided the conceptual background to design and implement conceptual programming-based tools [43, 86], which facilitate the design of an ontology-driven conceptual modeling system with industrial support (e.g., Integranova, www.integranova.com).

At the same time, the BWW ontology has been criticized (e.g., [121], especially with respect to the assumptions underlying the ontology roots; that is, the philosophical beliefs of Bunge. Notably, BWW was developed on a subset of Bunge’s ontology [17, 18] which is now over 40 years old. Since the publication of these two primary sources for the BWW ontology, Bunge published over 100 books and 300 papers [31], in which his ideas were further expanded, refined, and sometimes altered. These additional writings lead to the following research questions.

Is there a need to revise the original BWW? Are statements such as “Bunge believes the world is made of things” still appropriate, given the evolution of Bunge’s work? Is an expansion of BWW needed [93] or do the ideas expressed by Bunge, which are not part of BWW, call for a new ontology? Can the initial tenets of this new ontology be formulated? What are the implications of such a new ontology for the development and use of IT?

To address these research questions, we first discuss the basic tenets of BWW to establish a common understanding of Bunge’s ideas. We then consider the more recent ideas of Bunge and present them as a proposed, new ontology, which we call Bunge’s Systemist Ontology (BSO). The new ontology is compared to BWW, the results of the comparison are discussed and implications for future research are detailed.

## Background: Bunge–Wand Weber Ontology

Yair Wand and Ron Weber offer a first-hand account [115] of their motivation to ground information systems research in a foundational ontology, as well as of how they developed a set of theories based on what became known as the Bunge–Wand–Weber ontology (BWW). The theories were: ontological expressiveness, a representation model, and a good-decomposition model. Although they consulted other sources, the primary foundation of BWW are two seminal manuscripts on ontology by Bunge [17, 18], which are part of his eight-volume *Treatise on Basic Philosophy*.

The BWW ontology [110, 111, 114] argues that the world is made of *things*—substantial individuals—which possess properties. Things may form composite things and interact with each other, leading to the acquisition of new properties or loss of existing properties. Properties are not directly accessible to human observers, resulting in the notion of attributes, which humans ascribe to things, but which may or may not be accurate or complete representations of the underlying properties. Sets of things form systems if, for any bi-partitioning of the set, coupling exists among things in the two subsets. The main constructs from Bunge as adopted into BWW are: thing, property, attributes, functional schema, state, law, state space, event, history, coupling, system, class, kind, and their derivatives (e.g., lawful state space) (see Table 1, p. 222 in Wand and Weber [113]).

The BWW ontology, as well as the theories, models and methods derived from it, has been used widely in conceptual, empirical and design work in information systems, conceptual modeling, software engineering and other areas [115], making it an important development in the area of ontology in IT [66]. Despite its influence [66, 88, 94], the ontology has been criticized for its narrow physicalist focus, lack of attention to social and psychological phenomena, and postulates which may be problematic for modeling certain types of domain rules. Examples are proscribed optional properties, denied independent existence of properties, and properties of properties [56, 76, 105, 121].

A generally overlooked issue is that the BWW ontology is based on only selected references from Bunge. Although there were some attempts to expand BWW to incorporate other ideas of Bunge [93], these were still narrow in scope and did not realize widespread adoption.

The basis for BWW is two, albeit seminal, manuscripts by Bunge. However, as Bunge frequently noted, ontology is inseparable from other beliefs, such as on how to acquire knowledge in the world [26]. Indeed, the *Treatise* contained many additional beliefs, related to semantics, epistemology, methodology, ethics, and technology. During the 40 years since the publication of the 1977 and 79 volumes, and even since the last book of the *Treatise* on ethics [21], Bunge published over 400 manuscripts, in which his ideas were further expanded, refined, and sometimes, altered.^2^ Some of these more recent ideas are of great potential relevance to IT, because they directly dealt with issues of information technology (e.g., [30].

## Fundamentals for constructing Bunge’s Systemist Ontology

The task of understanding the differences between Bunge’s ideas enshrined in BWW and his other, and more recent thinking, meets a challenge: the ideas which comprised BWW were carefully distilled, whereas the more recent ideas were not. Although based on two volumes, BWW was founded on a self-contained *Treatise on Basic Philosophy* which developed and presented ideas systematically and with great internal consistency. These began with semantics [16], then ontology [17], followed by epistemology [19], methodology [19] and ethics [21]. In contrast, Bunge’s works since the *Treatise* (1974–89) are not assembled into a dedicated, self-contained single compendium. Rather, it is a collection of over 400 essays, papers and books [22, 26, 28–30], which require dedicated synthesis.^3^

To address our research questions, we, thus, engaged in a comprehensive and systematic effort to catalog and distill these beliefs. This project was conducted over five years (2015–2020) and includes the last known publication by the late Bunge.

First, we began to assemble a library of publications by Bunge and conducted a scoping survey of his writings to gain a preliminary understanding of the extent of the modifications and expansions compared with BWW. Second, half-way into the process, the first author of this paper contacted Mario Bunge, who kindly agreed to meet and presented a general overview of his earlier and most recent thinking, answering numerous clarifying questions. Third, we reviewed all pertinent publications using *Google Scholar* and Bunge et al. [31] as sources.^4^ Fourth, we followed the logical path outlined in the *Treatise* (i.e., ontology, epistemology, methodology and ethics) as re-iterated and explained by Bunge in other sources (e.g., [26] to catalog the ideas. We began with basic assumptions about reality, followed by the problem of knowledge of reality, and then the application and use of knowledge in society (e.g., in policymaking, science and daily life). Fifth, we began synthesizing the ideas, favoring the most recent publications (e.g., [28, 29] and referencing earlier publications (e.g., [26], Bunge’s own memoirs [27], and authoritative studies on Bunge [31], for clarification or expansion of ideas, as needed.^5^

The intended result is a systematic synthesis of Bunge’s publications aimed at distilling and presenting a single, coherent and consistent set of beliefs with the aim of using these ideas within the context of information technology. Bunge kindly clarified some of the ideas of his ontology and also shared a copy of his unpublished manuscript.^6^ However, all claims made here are justified either through direct references to published works by Bunge or are explicitly noted as our inferences and derivations.

To report the findings, we analyze the constructs of BWW [Table 1, p. 222 113] and compare them to what we coin as *Bunge*’*s Systemist Ontology (BSO)*. The BSO captures broader and more recent set of ideas developed by Bunge. Indeed, Bunge uses multiple labels to describe his set of beliefs (e.g., “emergentist materialism” [25], “hylorealism” [26, p. 27]), but the most frequently used term appears to be “systemism” [18, 24, 29], thus giving the name to the new ontology. This label was also confirmed to be preferable by Bunge himself during our interactions with the philosopher-physicist.

## Understanding Bunge’s recent works

We first compare BSO with BWW by focusing on the constructs they have in common. Since BSO is broader than BWW, we also provide an overview of the constructs in BSO that extend beyond those of BWW.

### Bunge’s Systemist Ontology versus BWW

The BSO claims *reality* is all that we know to exist and distinguishes five “kinds” or “levels” of *reality*, including physical, chemical, biological, social and technical [22, p. 25]. One level emerges from another (e.g., social from biological) via emergent properties (discussed later) and higher levels are grounded in the underlying physical level.

The BWW ontology postulates that *reality* is made of *things*, which have properties [17], pp. 26–29). Things are “substantial individuals,” which could be *composed* of other individuals or be *simple*, structureless and atomic [111, p. 126]. However, many things also form systems, which have things as their components. Hence, Bunge poignantly titled his 1979 volume of the Treatise, “Ontology II: A World of Systems” [18].

In his most recent writings, Bunge put forward a more intriguing idea: every *thing* is likely a *system*, which we deem an essential claim of BSO. In BSO a system is the ontological primitive. Per BSO, *the world is made of systems*. What precipitated this change for Bunge and what is its basis? We suggest the postulate “the world is made of systems” is grounded in three more recent beliefs of Bunge.

First, using *the notion* of a system allowed Bunge to reason about entities for which the notion of a *thing* was either ontologically inapplicable with respect to modern scientific knowledge (e.g., consider photon’s wave-particle duality), or linguistically awkward. Bunge [28] explains (p. 174):

The word ‘system’ is more neutral than ‘thing’, which in most cases denotes a system endowed with mass and perhaps tactually perceptible; we find it natural to speak of a force or field as a system, but we would be reluctant to call it a thing.

Second, Bunge, following recent advances in particle physics, became convinced that *there are no simple, structureless entities*. Bunge [28] explains (p. 174, emphasis added):

By calling all existents “concrete systems” we tacitly commit ourselves *in tune with a growing suspicion in all scientific quarters* - that there are no simple, structureless entities.

Bunge notes that the history of science teaches us that things once thought to be irreducible and fundamentally simple (e.g., atom) have later proven to be complex. Bunge asserts that simple and structureless things, if exist at all, exist only at the quantum level [24, p. 148]:

Only particle physicists study non-systems, such as quarks, electrons, and photons. But they know that all such simple things are parts of systems or will eventually be absorbed by some system.

Thus the idea that “there are no simple, structureless entities” is not only an ontological, but also a normative belief: “[t]his is a programmatic hypothesis found fertile in the past, because it has stimulated the search for complexities hidden under simple appearances” [28, p. 174]. It may very well be that the elementary particles of today (e.g., quarks, bosons) presently considered atomic, in time can be found to be complex. In numerous of his writings, Bunge stresses that he views his ontology, not only as a theory of what exists, but also as a normative template for the kinds of questions to ask when inquiring about the nature of reality [22, 26, 27].

Third, systemism for Bunge offered a more balanced approach for *describing reality* (an idea of especial interest to conceptual modeling in IT). For Bunge, systemism holds numerous advantages, as it conceptually lies between individualism (which under-represents internal structures of a system, its relationship with the outer environment, its levels of composition and emergence) and holism (which is not interested in the components and specificity of subsystems). Systemism represents the best of these two ideas, without sacrificing the benefits of each [24]. This is how Agazzi, a friend and close associate of Bunge, summarizes his views, which he debated with Bunge extensively [1]:

[Bunge] explicitly presents his position (which he calls “systemism”) as intermediate between two erroneous extremes, “atomism” and “holism”. The weakness of atomism resides in that it ignores the relevance of properties and especially relations, without which it is impossible to distinguish a single “aggregate” from a “system”. The weakness of holism resides (according to Bunge) in its pretension that the knowledge of the whole must precede and make possible the knowledge of the parts. Systemism avoids both mistakes by recognizing that the whole “results” from the correlation of its parts and at the same time has influence on their functioning.

Thus, the tenet that “the world is made of systems” is an ontological hypothesis and a normative postulate. It offers interesting possibilities for modeling in IT, as discussed later. However, it also offers a notable challenge. Indeed, it could be possible that there are no simple, structureless entities and that even elementary particles may be systems (i.e., composed of other systems), yet this possibility implies an infinite recursion. Within the context of IT, we suggest two ways to address this problem, while simultaneously providing the foundation for future studies to conduct a dedicated analysis of this issue.

First, the majority of extant applications of IT deal with domains beyond the domain of elementary particles and quantum physics. For example, the typical use cases of systems analysis and design such as ERP, social media, e-commerce, personal productivity software, deal with entities such as customers, suppliers, orders, social media friends. These entities are indeed systems and are composed of other systems which in turn are composed of other systems. This is an important realization, because it liberates such applications from the need to resolve the fundamental ontological status of the “component” or “system part” and deal with the possible infinity of subsystems.

Second, some applications do engage with elementary particles and may involve modeling entities, for which there is no presently known structure (e.g., quarks, bosons) [97]. For these cases, we suggest using Bunge’s construct of a system, but not showing the components of it. Indeed, as Bunge suggested, the notion of a thing would not suffice for some of the entities in this domain (e.g., forces, fields, photons). In such an approach, the construct of a system is, not only a construct of convenience, but also a hypothesis based on the most recent speculation of Bunge that such elementary particle may, indeed, have structure that could be discovered later. Thus, adopting BSO within the context of IT allows us to potentially remove the notion of a thing, simply replacing it with the system construct.

Having established the basic tenet of BSO, we now consider the basic notions related to systems. In the *Treatise*, Bunge postulated that any system should have “a definite composition, a definite environment, and a definite structure. The composition of the system is the set of its components; the environment, the set of items with which it is connected; and the structure, the relations among its components as well as among these and the environment” [18, p. 4].

In later writings, this initial idea was developed into a Composition, Environment, Structure and Mechanism or *CESM model*. In CESM in addition to the composition, environment, and structure (present in BWW), Bunge added “mechanism” [24]. Mechanism is defined as “characteristic processes, that make [the system] what it is and the peculiar ways it changes” [26, p. 126]. The CESM model is a principal model of systems in BSO, which can be used to reason about and describe systems. To illustrate, Bunge provides an example of a traditional nuclear family—a type of a social system [26, p. 127]:

Its components are the parents and the children; the relevant environment is the immediate physical environment, the neighborhood, and the workplace; the structure is made up of such biological and psychological bonds as love, sharing, and relations with others; and the mechanism consists essentially of domestic chores, marital encounters of various kinds, and child rearing. If the central mechanism breaks down, so does the system as a whole.

The inversion of the relationship between things and systems, and the potential obviation of the need for *things* in BSO, represents a major change, as the construct of thing has been a founding one for BWW and has been the conceptual foundation for many studies that adopted BWW [72, 83, 86, 108]. However, *things* in the social and technical levels of early Bunge were effectively systems [18]. This change can be easily accommodated by much of the prior work that used BWW with a mere replacement of a label.

As in BWW, BSO upholds beliefs about the relationship between systems and properties. Systems have properties. Properties do not exist outside of systems [28, p. 175]: “Property-less entities would be unknowable, hence the hypothesis of their existence is untestable; and disembodied properties and relations are unknown.” As in BWW, properties according to BSO do not exist in themselves: “However, … can be material only derivatively…: there are neither properties nor relations in themselves, except by abstraction.” [26, p. 11].

Notions of classes and kinds are used in BSO. In BWW, classes are sets of things sharing “a common property”, whereas *kinds* are sets of things which share “two or more” properties [113, p. 223]. Systems with “one of more” common properties in BSO [22, p. 111] form *classes* and those with properties which are interrelated, form *kinds* [26, p. 13].

The emphasis on systems carries other implications, as this new postulate is propagated throughout Bunge’s recent works. According to BSO, some, but not all (an important caveat), systems undergo change, resulting in emergence (addition of new) or submergence (loss of old) of properties. To account for this situation, BSO continues to use the construct of *state*. Bunge [28, p. 171] defines *state* as “the list of the properties of the thing at that time.” This definition is similar to that of BWW [17, p. 125]. A state can describe multiple properties (at the same moment in time) [26]. A given system has the properties of its subsystems, as well as its own, termed *emergent properties* (an idea unchanged since BWW), but now gaining greater focus in BSO, as a key implication of systemism.

In BWW, there are postulates that deal with changes of states (i.e., events) and how the properties that make up the states are perceived by humans (i.e., attributes) [17]. Whereas BWW applied the notion of a state to all things [17, p. 123], per BSO, Bunge [26] makes an important distinction between systems which undergo change and those that do not. Per BSO, Bunge distinguishes two kinds of system: *conceptual* and *concrete* [22, p. 270]. A *conceptual* (or formal) system is a system all the components of which are conceptual (e.g., propositions, classifications, and hypothetico-deductive systems-i.e., theories). This is contrasted with *concrete* (or *material*) systems which are made of concrete components (i.e., subsystems, such as atoms, organisms, and societies), and may undergo change.^7^

What distinguishes concrete and conceptual systems is the essential property of *mutability*, as a key element of BSO, which *only concrete systems possess*: “mutability is the one property shared by all concrete things, whether natural or artificial, physical or chemical, biological or social, perceptible or imperceptible” [26, p. 10]. Bunge thus explains that changes in systems may only occur if the systems are concrete [26, p. 11]:

heat propagation, metabolism, and ideation qualify as material since they are processes in material things. By contrast, logical consistency, commutativity, and differentiability can only be predicated of mathematical objects.

Concrete systems change in the virtue of energy transfer. For Bunge, “the technical word for ‘changeability’ is energy” [26, p. 12], such that:

To repeat, energy is not just a property among many. Energy is the universal property, the universal par excellence.

We, thus, obtain a more formal definition of a *concrete system* in BSO as a system that has energy [26, p. 12].

In BSO, when systems interact, they transfer energy from one to another. Bunge dedicates considerable time to the notion of energy. He considers different kinds of energy, including mechanical, thermal, kinetic, potential, electric, magnetic, gravitational, chemical (e.g., in [26]. Energy transfer leads to change in states of things, as they acquire or lose their properties. This produces events and processes. Energy when paired with *artificial code* (instructions which correspond to ways to understand meaning) may transmit *information;* that is, carry meaning for an observer. This idea is not found in BWW, but of special relevance to information technology.

In contrast to concrete systems, conceptual systems do not change since they, themselves, do not possess energy. Naturally, in thinking about and communicating conceptual systems, energy transfer occurs. However, this energy transfer occurs within and between concrete systems (i.e., humans who are thinking and communicating these ideas). Bunge suggests that per se, conceptual systems do not harbor energy. They are mental tools that humans use to reason about concrete and other conceptual systems. Conceptual systems cannot transfer energy from one conceptual system to another. Conceptual systems, therefore, do not change per se; what changes is the knowledge of them in the mind of the observer (i.e., a concrete system). One conceptual system can be replaced by another when the latter is found to be more useful, convenient or expedient in some other way (e.g., simpler to remember or learn).

The consequence of the re-definition of systems as either energy-bearing or not, implies another change compared to BWW. Thus, whereas in BWW an event has been understood as a “change in state of a thing” [113, p. 222], in BSO, an event is understood in terms of energy, thus being applicable only to *concrete systems.*^8^ Bunge views event as an energy-involving construct [26, p. 91]:

Event C in thing A causes event E in thing B if and only if the occurrence of C generates an energy transfer from A to B resulting in the occurrence of E.

Multiple events form *processes*, defined as “a sequence, ordered in time, of events such that every member of the sequence takes part in the determination of the succeeding member” [28, p. 172].

The demarcation between events applicable to concrete versus conceptual systems affects the definition of the notion of *law*, which is now applicable to concrete systems only. Laws are stable patterns which hold “independently of human knowledge or will” [22, p. 27]. In BSO, *conceptual systems* do not obey laws, but rather obey rules of logic or other considerations imposed by humans who create or use these systems [26].

### BSO beyond BWW

Although Bunge considered himself an ontologist, for him the connection between ontology and epistemology was inseparable. Notably, however, issues of ontology, epistemology, methodology and ethics were separated into standalone volumes in the *Treatise*. This could potentially explain why BWW focused on the constructs related to material reality. In recent writings, Bunge enmeshes the discussion about systems and their properties with epistemological issues within the same volumes. As a result, in BSO, the connection between his ontological beliefs and his beliefs about the nature of knowledge of reality becomes explicit.

In BSO, an event or a process as it appears to some human subject is termed *phenomenon* [28, p. 173]. It is an occurrence registered by the sensory apparatus of humans or other animals triggered by a change or a series of changes in the state of a concrete system. For example, the sensation of wind blowing in the face or an act of watching YouTube videos produce a complex chain of biochemical reactions in humans who experience these *events*. These sensations are produced by the interaction between systems external to the human observer and the human observer (who is a system also) [22]. Phenomena, therefore, are special kinds of energy transfer, present when sentient beings are interacting with the world. Phenomena may arise due to direct interaction with physical systems (e.g., pressing an elevator button) or indirectly (e.g., via a signal or information). Phenomena are always “in the intersection of the external world with the cognitive subject” [28, p. 173].

Events, processes, phenomena, and concrete systems are material instances of the mental concept of *fact.* That is, they lie “in the extension of the concept of fact” [28] p. 174]. Thus, Bunge uses the notion of *fact* (which is an epistemological construct) to group important related ontological constructs that have special relevance to humans. Facts for Bunge are kinds of *objects:* “whatever is or may become a subject of thought or action” [28, p. 174]. What makes them special compared to other types of objects is that facts are “known or assumed – with some ground – to belong to reality” [28, p. 171]. It does not appear that Bunge seeks to demarcate reality from non-reality (Bunge believed in a single world). Rather, Bunge indicates that all objects belong to reality, with only facts representing specific, important aspects of systems. Through the fact construct, BSO connects the fundamental ideas concerning the composition of reality to the mental world of humans.

Bunge asserts that phenomena are merely small fractions of the *facts* constituting the object of an investigation. This makes Bunge equate phenomena with “observable facts;” that is, the facts that can be sensed directly. As BSO states, “the observable facts or phenomena are data suggesting or confirming the existence of more interesting facts behind” [28, p. 177].

It is a subject of centennial debates in philosophy whether human observers have access to more than just phenomena. The position of the *phenomenalism* holds that only direct sensations and experiences are knowable [64]. In contrast, various strands of *realism* generally posit that reality beyond sensations can be known [61]. This can be accomplished with the aid of experimentation, theory testing, imagination and logical inference. Bunge is a proponent of the latter [28]. For Bunge [28], the pragmatic benefit of realism is that it encourages thinking and action beyond sensations and motivates an active, inquisitive stance toward reality.

For Bunge, facts are iceberg-like in that they are largely submerged under the surface of immediate sensory experience. Furthermore, the phenomena are often quite different from the concrete systems upon which they are based. An example is the difference between the visual sensations caused by a flash of lightning compared with the actual chemical and electric other physical processes involved in the unraveling of this concrete system.

In both BWW and in BSO, Bunge distinguishes between properties and attributes. However, it is BSO that offers an expanded explanation for what constitutes an *attribute.* An attribute is a mental concept (i.e., an object of thought), which may correspond to phenomena. When we, as humans, experience lightning, we experience a bundle of properties associated with this complex concrete system. However, not all sensory experiences related to lightning have associated attributes. Thus, we may label lightning as “bright” and “dangerous”, but generally do not have an established attribute to describe specific smells associated with lightning. In other words, certain properties of systems may be experienced as phenomena, with some of the phenomena grouped into attributes human find useful. However, not every attribute can be traced to an underlying property. Because attributes are mental objects, Bunge admits there is a possibility of humans having attributes that may not correspond to any underlying physical properties of material systems (e.g., “magical” is an attribute of a shield of a fictitious hero). Thus, not all attributes are grounded in phenomena.

Bunge extensively deals with non-observable facts or what he calls, “submerged” facts. For Bunge, they are especially interesting because they underscore the value of science and scientific thinking for humans. Since most reality is inaccessible to direct observation, it must be hypothesized. A *hypothesis* is a conjecture about the relationship between the observed and the unobserved facts [28]. A hypothesis need not be a scientific one. Humans routinely hypothesize, without being consciously aware of doing so. For example, when looking out of the window, we may observe clouds forming in the sky. Doing so may lead us to take an umbrella when we venture outside. These physical events are linked with a number of hypotheses about the relationship between facts about systems.

For Bunge more interesting hypotheses are those which require extensive elaboration and thinking. These types of hypotheses, although still present in day-to-day life, are most commonly found in science. To test such hypotheses, definite relationships between the unobserved and the observed facts must be developed, by which the observed can count as evidence for, or against, the existence of the hypothetically unseen, and the unseen can explain what can be seen. These relationships are represented by hypotheses and *theories* [28, p. 177].

To reason about deeper levels of reality, one needs to connect phenomena with unobserved systems. Hence, *observation* becomes a key construct of BSO at the nexus of ontology and epistemology. Observation is defined as “purposeful and enlightened perception” [28, p. 181]. It is purposeful or deliberate because it is made with a deliberate goal and enlightened because it is guided by prior knowledge of the observer. The object of observation is a *fact* in either the external or the inner world of the observer. The former, for example, can be the sight of an approaching passer-by, whereas the latter could be thoughts, memories, and mental images that are available to the observer through introspection.

The subject or observer includes, of course, their perceptions. The circumstances of observation are the environment of the object and subject. Both the observation media and the body of relevant knowledge are means for the observer, but not for the instrument designer or for the theoretician. Observation statements have the following form: “w observes x under y with the help of z”.

There is no “end” to the BSO per se. Recall that BSO is not published in a self-contained treatise. Bunge continuously stresses the interdependency between ontology and other beliefs. Indeed, Wand and Weber engaged with other ideas of Bunge, as did other scholars (e.g., [79], [93]), and acknowledged the existence of other constructs and more recent beliefs. As they note, Bunge “has written extensively about social phenomena using constructs based upon his ontology” (e.g., [23], [115]). Yet, much of the IT community adopted the views of Bunge stemming from BWW, making this an important benchmark comparison.

### Similarities and differences between BWW and BSO

Based on the exposition of BSO, which captured more recent beliefs by Bunge in comparison to BWW, we draw the comparisons summarized in Table 1.

**Table 1:** Comparison between foundational constructs of BWW and BSO*

Construct	Definition from BWW*	Definition from BSO	Comparison and Analysis
Thing	“A thing is the elementary unit in our ontological model. The real world is made up of things. A composite thing may be made up of other composite things or primitive things”	N/A	In BWW thing is the fundamental ontological primitive which stands in its own. In BWW a system is a kind of a thing—a thing that has structure. In BSO, we suggest all things to be systems (note our caveat re elementary particles explained above)
System	“A set of things is a system if, for any bi-partitioning of the set, coupling exist among things in the two subsets”	“complex object every part or component of which is connected with other parts of the same object in such a manner that the whole possesses some features that its components lack—that is, emergent properties” [22, p. 20]	In BWW system is understood in terms of things - the fundamental ontological primitives. In BSO, thing is defined in terms of a system, a thing is a kind of system
Property	“Things are known via their properties. A property maps the thing into some value”	The substance (matter and energy) that make concrete systems what they are and predicates of conceptual systems [28, p. 175]	Neither BSO nor BWW have formal notions of property. Bunge’s recent writings (e.g., [28, p. 175]) re-iterated his early ideas that properties do not exist in themselves and property-less entities also do not exist. The new notion of energy in BSO gives the property concept more formality, albeit it only applies to concrete systems
Emergent property	“A property of a composite thing that belongs to a component thing is called a hereditary property. A property that does not belong to any of the composing things is called an emergent property”	“To say that P is an emergent property of systems of kind K is short for “P is a global [or collective or non-distributive] property of a system of kind K, none of whose components or precursors possess P” [25, p. 25]	Emergent property has undergone a shift from BWW to BSO, wherein the latter ontology defines it as property of systems
State	“The vector of values ​​for all properties of a thing is the state of the thing”	“list of properties of the [system at a given instant of time]” [28, p. 171]	In BWW and BSO state has the same meaning
History	“The chronologically-ordered states that a thing traverses in time are the history of the thing”	“a sequence of states [of a system]” [22, p. 24]	Same notion, only applied to systems
Subsystem	“A subsystem is a system whose composition and structure are subsets of the composition and structure of another system and whose environment is a subset of the environment of the other system in union with the things that are in the composition of the other system but not in the composition of the subsystem”	“[system] is “both a system and part of another system” [22, p. 270]	The construct is the same in BWW and BSO. Note, BWW’s version is consistent with the systemist approach (i.e., subsystems are systems)
Event	“An event in a thing is a change of state”	“Event C in thing A causes event E in thing B if and only if the occurrence of C generates an energy transfer from A to B resulting in the occurrence of E” [26, p. 91]	The construct is the same in BWW and BSO. Note the inconsistency in BSO, as event is still defined in terms of things, rather than systems
Class	Set of things sharing “a common property”	Systems with “one of more” common properties [22, p. 111]	Notable change in BSO of conceptualizing classes as (conceptual) systems
Kind	Set of things which share “two or more” properties	Classes with properties which are interrelated [26, p. 13]	A change in BSO which stipulate kinds to have interrelated properties—a notion more consistent with definition of natural kinds by other researchers [48, 60]
Process	N/A	“sequence, ordered in time, of events such that every member of the sequence takes part in the determination of the succeeding member” [28, p. 172]	New construct in BSO
Phenomenon	N/A	“is an event or a process such as it appears to some human subject: it is a perceptible fact” [28, p. 173]	New construct in BSO
Fact	N/A	“whatever is the case, i.e., anything that is known or assumed - with some ground - to belong to reality” [28, p. 171]	New construct in BSO
Object	N/A	“whatever is or may become a subject of thought or action” [28, p. 174]	New construct in BSO
Observability	N/A	“x is observable only if there exist at least one recording instrument w, one set of circumstances y, and one set of observation tools z, such that we can register x under y helped by z” [28, p. 185]	New construct in BSO
Observation (direct observation)	N/A	“purposeful and enlightened perception: purposeful or deliberate because it is made with a given definite aim; enlightened because it is somehow guided by a body of knowledge” [28, p. 181]	New construct in BSO
Observation (indirect observation)	N/A	“hypothetical inference employing both observational data and hypotheses” [28, p. 181]	New construct in BSO
Observer	N/A	“subject [of observation]” [28, p. 184]	New construct in BSO
Hypothesis or factual hypothesis	N/A	corrigible proposition about yet unexperienced or in principle unxperientable facts [23, p. 254]	New construct in BSO
Theory	N/A	“a system of propositions some of which are hypothesized and the remainder of which are deduced from the former” [22, p. 113]	New construct in BSO

*Note, the comparison is based on constructs from BWW as provided in Wand and Weber [113], pp. 222–223). Some constructs of BSO (e.g., process, fact) have been part of the *Treatise*, but were not included in that original source for BWW

First, it is evident that more recent thinking by Bunge remains partially consistent with BWW. Table 1 compares BWW and BSO, demonstrating that many ideas in BSO are the same as in BWW. These include the notion of things, properties, events, attributes, classes, laws. The relationships between many constructs remain the same (e.g., properties and attributes, properties and things). Thus, BSO carries many of the same design implications for IT, as does BWW. Included is the denial of the existence of properties, which has known implications for conceptual modeling research, such as problems of optional properties or properties of properties [12, 13, 34, 52], emergence, and lack of direct human access to reality (i.e., to the properties of systems).

Second, many of the changes introduced by BSO could be handled by appropriate qualifications or more precise specifications of the already existing notions (e.g., that concrete systems undergo change via energy transfer, but conceptual systems do not). The notions of *things* and their properties are still present because some systems can be viewed as systems for which no structure is modeled (as we discussed earlier). The relationship between properties and attributes can now be understood by the notion of *phenomenon*. Notably, however, as the example of properties and attributes demonstrates, in some respects, BWW can be considered a subset of BSO, which abstracts from the richness and nuances of BSO but has greater parsimony.

Thus, there is an important continuity between BSO and BWW. This continuity is critical for assessing the status of impressive theoretical, conceptual and design research that stemmed from the ideas of Wand and Weber [111, 113, 115]. Hence, BSO could still be used to posit that classes “tyrannize” instances [83] or that optional properties should be proscribed (we leave the issue of whether such design proposition is appropriate for conceptual modeling outside our discussion). However, in BSO, this is true for concrete systems only, since conceptual systems do not follow the same principles as concrete ones do.

As with BWW, BSO continues to adhere to the tenets of scientific realism and grounds thinking into interpretation of the state-of-the-art knowledge in physics and other disciplines. The two ontologies are products of conceptualizing and synthesizing knowledge about the nature of reality as derived meticulously from what Bunge, as a physicist [15], assumed to be tenets of science. This makes the two ontologies especially valuable, as they promise to ground representations of reality based on these ontologies into solid scientific beliefs, thereby attempting to realize repeated calls of researchers to ground IT into deeper, more fundamental foundations [84, 107].

On the other hand, BSO covers more compared with BWW. We suggest, BSO is not an expansion of BWW that could be achieved by simply adding epistemology to BWW. Rather, BSO suggests a new way of thinking about reality. Furthermore, the basic tenet of BSO—the world is made of systems—departs remarkably from BWW. Hence, BSO embeds ideas about systems at its very core, taking it as its fundamental premise. As Bunge writes, this is not only an ontological, but also a normative stance [28]. It impels the users of his ontology to proactively seek complexity beneath the seeming simplicity. It is in this complexity that Bunge’s sees a path for uncovering the fundamental nature of reality. This is a core idea around which other elements revolve.

Furthermore, in BSO, Bunge made a concerted effort to shift the focus from material things to physical, biological, social and mental systems. This is evidenced by the many new constructs that are not part of BWW (Table 1). BSO, much more than BWW, is concerned with the relationship between physical and mental phenomena, advancing numerous novel constructs, such as observer, observation, hypothesis, theory, and fact.

BSO further deepens the understanding of the relationship between fundamental constructs of BWW, such as classes, kinds and things, and properties and attributes. In all cases, in order to gain a deeper understanding of this relationship, a closer examination of the mental world of humans was required. Although not examined in this paper in detail, Bunge also discusses social reality at length, including engaging with ideas of Searle [96]. For example, Bunge [22] makes contributions to social ontology, which has been cited as a limitation of BWW (e.g., [76].

When comparing BWW with BSO, we can also use the analogy of comparing classical physics with quantum physics. Classical physics is applicable to macroscopic particles, providing a coarse-grained perspective of reality, but hiding the microscopic world dimension that quantum physics analyzes. Since classical physics can be derived from quantum physics in the limit that the quantum properties are hidden, BWW can be considered a simplification of the BSO when BSO’s systemist perspective is reduced to material systems the internal complexity of which is abstracted away. We can then interpret BWW as the beginning of a fundamental way to represent reality. The BSO concepts provide a more complete and refined knowledge of reality.

Considering the differences between earlier and more recent thinking of Bunge, we therefore propose *Bunge’s Systemist Ontology* or BSO, as a new ontology, and a new, practically applicable, addition to the theoretical toolbox of IT.

## Implications of BSO for Areas of IT

The comparison between BSO and BWW implies that the broader and more recent ideas of Bunge carry exciting implications for ontology-based IT research and practice.

### Reinvigorating ontological debates in IT

BSO contributes to the long-standing research on using Bunge’s ideas as theoretical foundations for IT. Bunge is among the most influential ontologists for the fields of conceptual modeling, systems analysis and design, and software engineering research. Bunge’s ideas were, not only at the core of BWW, but also used widely in design and empirical studies on conceptual modeling [45, 82, 86, 118], business process modeling [10, 89], information quality [72]; Lukyanenko Wiersma, et al., [74, 109], data modeling and database design [83, 108], 1999), software engineering [85, 92], information systems requirements [65, 98, 106], and ontology engineering [5, 7]. Bunge’s ideas have also been frequently used as a benchmark for other ontologies and when analyzing the value of ontologies for IT [54, 56, 121].

Our research considers whether the ideas as expressed by BWW capture the most recent thinking of Bunge. As our work suggests, Bunge makes broader and deeper contributions than previously recognized. Although there is an overlap between BWW and BSO, BSO contains many new ideas, and hence carries new implications for the assessment of the applicability of Bunge for various disciplines of IT.

Future research could further examine the benefits and limitations of Bunge more broadly (i.e., BWW and BSO) for conceptual modeling, information quality, software engineering, and other areas which have thus far benefited from the exposure to Bunge’s thought.

### Supporting modeling in new domains

With a new way of conceptualizing reality and additions of epistemological constructs, BSO should be able to support design and use beyond that of BWW. Using the iceberg metaphor for representation of reality enables a specific example to be discussed. BWW provided the ontological basis of a conceptual programming approach called “OO-Method” [43, 86], together with its associated industrial tool, Integranova. Conceptually, OO-Method focuses on organizational systems and their associated database-based applications. It works well within this context, but, by considering BWW as the tip of the *reality representation iceberg*, several other systems appear to fall out its natural scope (e.g., deep learning reasoning, machine learning algorithms, AI conceptual applications as Explainable AI). With the ontological commitment provided by the “basic” BWW, it is very difficult to go beyond the notion of an organizational system (taken from the FRISCO Manifesto, IFIP WG 8.1. [46]), that is a type of concrete system well-characterized by using the “thing” concept of BWW. BWW can work well for representing the components of these concrete (material) systems, but it has difficulty representing conceptual systems (i.e., conceptual components of those organizational systems, as algorithms, functions, theories).

For BSO, that hidden part of the reality representation becomes accessible, especially through the explicit distinction between concrete and conceptual systems, and the coverage of reality beyond sensations, as well as the consideration of facts as being largely submerged under the surface of immediate sensory experience. All of these notions provide a way to understand the conceptual fundamentals of deep learning, machine learning, and any other conceptual (not concrete) system that BSO distinguished explicitly. For instance, applying BSO to neural networks could be considered as a connection between a concrete system (composed by other systems) and a conceptual system. An example is a neural algorithm that takes an image (for computer-based neural networks) or a perception (for human-based ones) of a concrete system as input and determines what systems are present in the image/perception as output.

The many new ideas of Bunge represented in BSO can provide a strong ontological foundation for many emerging applications in IT. To illustrate, we suggest two notable examples of potential applications of BSO into domains of applied machine learning based on complex models, such as deep learning and into explainable artificial intelligence.

Deep learning applications could benefit from BSO as this domain is based on a strong conceptual (but not material) basis. Deep learning is a type of machine learning that uses neural networks with multiple hidden layers, resulting in highly complex, but also very powerful models [11]. These models tend to capture what mostly corresponds to the tacit knowledge humans possess. This task requires the use of purely conceptual notions (high-level semantic variables) whose representation and reasoning capabilities form the basis for natural language communication and express algorithmic knowledge in software [6]. Hence, it is difficult to reason about the systems based on deep learning on the basis of a purely materialistic ontology. In contrast, BSO has more nuanced conceptual constructs, such as unobservable facts, hypotheses, observation and, broadly, the notion of hidden versus observable. These all appear to be of value for reasoning and modeling deep learning applications, as a future area of research.

Explainable artificial intelligence (XAI) is a socio-technical challenge due to the growing need to allow a machine to precisely explain a taken decision [40, 58]. It is, therefore, crucial to obtain a shared understanding of the domain under consideration (Lukyanenko et al. [68, 69], what again requires a conceptualization process that is hard to achieve using only the concrete (material) fundamental background that the original BWW provides. On the contrary, BSO provides constructs to support the characterization of a conventional XAI-based process [99]. Here, after designing the shared conceptual model, the task must be understood, the right scope selected, and the right data collected and its quality improved. In addition, the AI techniques that deliver results must be selected, in order to generate good explanations that adequately evolve over time. All of this is challenging and, obviously, requires future research, which could benefit from the idea captured in BSO.

### Evaluation and development of modeling grammars

The new constructs of BSO can also become valuable in evaluation (and possibly, development of new) conceptual modeling grammars. To illustrate one opportunity, recall that in BSO systems can be material or conceptual. For BSO, a critical demarcation is the absence of presence of *energy*, and the nature of energy exchange between systems. The nature of energy is a new consideration for conceptual modeling research and practice. As Bunge [26] argues, depending on the type of energy transfer, different interactions among systems become possible. If we model systems using, for example, classes in the UML grammar, the interaction among classes can be modeled using the association construct. However, this construct does not distinguish the types of energy that is being transferred during the interaction among the objects (instances of the classes).

**Fig. 1:**
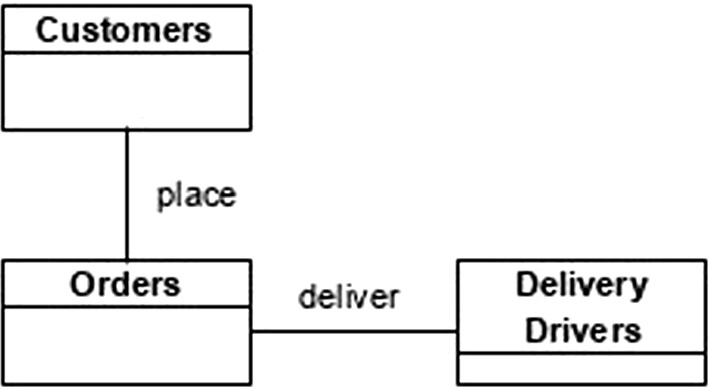
Fragment of a hypothetical UML diagram in an online order delivery domain

Consider an example of an online order delivery domain (with a fragment of a possible diagram shown in Fig. 1 in which real-world complexities are abstracted away). The diagram, which uses a UML notation, represents a domain with three kinds of real-world systems from the point of view of BSO: customers, orders and delivery drivers (with their internal complexity abstracted away for the purposes of the illustration). Their interactions are shown using the association construct. However, the nature of the interaction differs due to the different kinds of energy being transferred. In the case of a customer placing an order, it, presumably, involves the use of a mobile app over the Internet. In the case of a delivery driver, it involves an actual physical displacement, frequently at a considerable distance, guided by the mobile app and supported by other machinery and tools, which, in turn, also consume specific kinds of energy. Thus, the energy flow and energy requirements of the two associations differ in remarkable ways. Recognizing these differences could lead to a different appreciation for the kinds of resources needed to enact, manage and support the interactions of these systems. Many kinds of valuable inferences can be drawn from the knowledge of the nature of energy transfer between the systems (e.g., that delivery drivers require fuel, whereas customers require a stable Internet connection and enough battery charge in their cellphones). Each of these inferences can prove beneficial for building effective information technologies which support and enable interactions of systems in this delivery domain.

The notion of energy is new to conceptual modeling research. Therefore, future studies are needed to explore the implications of this new idea. Modifications to existing modeling grammars may also be needed to allow constructs that represent associations among systems to capture the different kinds of energy transfer, if such capture proves to be valuable for modeling purposes.

### Support and guidance for novel design patterns

Some of the design implications of BSO, while not necessarily requiring modifications to modeling grammars, may be useful as best design practices or design templates. To appreciate this potential, consider the fact that BSO provides several new ontological constructs—ontological primitives, including observation, hypothesis, and fact. These constructs of BSO appear particularly useful for a variety of modern applications; for example, the observation construct can, potentially, be used to model scenarios where people observe some things in reality and post these on social media.

These ontological primitives will not likely require a modification to existing conceptual modeling grammars, as they can be modeled as classes, or entities using grammars such as UML, ER or ORM and incorporated into existing modeling grammars as *modeling patterns*. The notion of a modeling pattern has been adapted in conceptual modeling research [51] from the field of architecture and “describes a problem which occurs over and over again in our environment, and then describes the core of the solution to that problem, in such a way that you can use this solution a million times over” [2].

**Fig. 2:**
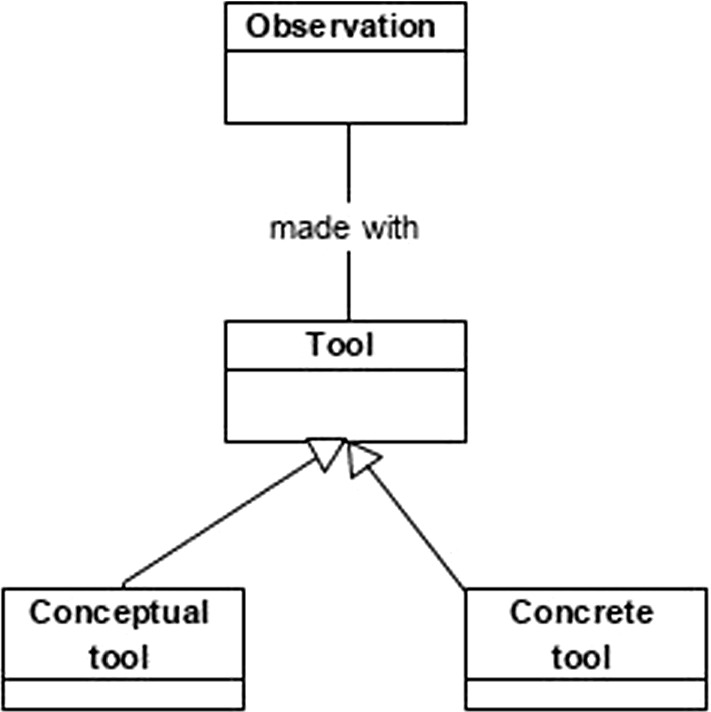
Pattern based on BSO illustrating typical observation made within a domain

Likewise, BSO can contribute a variety of new design patterns. Figure 2 depicts one such possible pattern using UML, in which an observation in a domain is modeled (again with real-world complexities abstracted away, for the purposes of the illustration). In addition to modeling the observation object itself, the contribution of BSO is to suggest modeling conceptual and concrete tools used to make the observation. Such pattern, for example, can be used for modeling social media applications where an observation can be an observation of a hotel (within the context of submitting a review). An app designed following the modeling pattern in Fig. 2 can also capture whether there are any photographs made along with the observation (i.e., corresponding to the concrete tool object), and also goals, assumptions, biases and intentions of the person making the observation (i.e., the conceptual tool). Such modeling pattern can facilitate the collection of more complete information, which allows for better interpretation of social media data. Future research can consider such design patterns, as well as propose and evaluate any new ones.

As another case of useful design patterns, BSO provides new and expanded expressiveness for modeling systems. Consider, for example, the relationship between things and systems. In contrast to BWW’s emphasis on *unique things*, BSO highlights the importance of representing structure, relationships between systems, emergence, levels, and interactions among subsystems. In BSO, Bunge clearly wishes to balance his views between the value an individual-focused perspective may bring versus a perspective that is more sensitive to the whole.

Information systems development, including conceptual modeling and user interface design, can incorporate Bunge’s CESM model (composition, environment, structure and mechanism) as a modeling pattern for describing systems and capturing user information regarding systems. Thus, a conceptual model of a domain can contain and represent elements consistent with CESM and incorporate them into conceptual modeling grammars as constructs. Then, for example, a project interested in recording data on some systems (e.g., sales, customers, markets, or natural phenomena, such as climate) could represent internal structures of the observed system, its relationship with its outer environment, its levels of composition, and the components and specificity of subsystems.

To illustrate further, consider a citizen science project [14, 36]; Lukyanenko et al. [75], which involves collecting citizen observations of lichens (a focal system of interest). Following BSO, the analysts could produce data collection interfaces and requisite database structures that capture: citizen observations of the structure of the lichens observed; the hosts to which the lichens are attached (its environment) and other external systems (e.g., the ecosystem); the individual strands that make up a collection of lichens (its components); and the properties of individual strands of lichens. By adopting the CESM modeling pattern or template, the citizen science projects can collect more complete data on systems of interest, thus increasing the potential of such data for insights and actions.

Many studies that follow early work of Bunge base modeling choices on the assumption of the primacy of individuals in logical database design, conceptual modeling grammars, information quality, and design collection processes [71–73, 83, 95]. Bunge extensively discusses the limitations of an individual-focused perspective and suggests that a more balanced approach—one that considers both individuals and collectives—may be more fruitful [22, 24]. BSO seeks to promote such balanced perspective, and thus can pave the way to even more expressive conceptual modeling grammars and IT designs realized in future studies.

### Work on formalizing BSO and evaluating its implications

Much work remains to study BSO in its own right, including formalizing BSO into a finite set of postulates (analogous to Wand and Weber [112]). Part of this effort should involve ensuring the final ontology is internally consistent; for example, BSO’s notion of event is defined based on things, rather than systems. The work should also continue investigating areas of IT practice that could benefit from the application of these ideas.

Although not directly engaging with conceptual modeling in IT, Bunge investigated issues of technology design and representations in science [16, 20, 30]. In these writings, he briefly considered the implications of his ontology for representing reality in artifacts, including social policy plans or architectural blueprints. Here, Bunge [20, p. 244] suggested that “a design or plan is *defective* if it overlooks any of the three features of any system: its composition, environment or structure (both internal and external)” (emphasis added).^9^ Thus, Bunge, himself, believed his ontology should be incorporated into design and action models and advanced an empirical claim that these models would be defective otherwise. This is a strong assertion that will require future research to corroborate or falsify.

## Conclusion

The philosopher Mario Bunge made a profound impact on the fields of conceptual modeling, software engineering, information quality, and database design. Much of this influence has been via the BWW ontology, which has made substantial contributions to the theory and practice of IT and conceptual modeling.

Recognizing that there are many concepts and ideas that have deep implications for understanding the reality that IT needs to model, we conducted a multiyear analysis of Bunge’s writings, which included personal consultations with Bunge. As a result, we gained a new perspective on the ideas and beliefs of Bunge. These ideas do not constitute a mere expansion of prior work. Rather, we synthesized the recent thinking of Bunge in the new ontology, the *Bunge Systemist Ontology* or *BSO*. Parts of BWW and BSO overlap precisely, so an important continuity between ontological work based on Bunge in IT is preserved. In addition, BSO promises new opportunities for IT as it orients the modeling efforts from individuals to systems, and ushers in much greater consideration of epistemology and axiology. Hence, a new ontology is warranted.

BSO contains concepts that can raise new prospects and possibilities for information technology, including for conceptual modeling, software engineering, ontology engineering and other areas of IT. We detail some of these possibilities in a set of research opportunities that focus on further discovering and applying the philosophical works of Bunge. In the world which deepens its reliance on IT, these new ideas of Mario Bunge could prove useful for further improving the way IT represents and shapes reality.

## Footnotes

1 A general ontology is thus different from a *domain ontology*. A domain ontology is a description (often formal) of constructs in a particular domain [78]. Examples include an ontology of Software Defects, Errors and Failures [41], database design [102, 103], or ontology of research validity (Lukyanenko et al. [70], and many others in diverse domains [78; 87].

2 An example of a reversal is Bunge’s admiration for Marxism-Leninism (an extensive set of beliefs transcending the general public’s most familiar ideas about politics and economy). It was Bunge’s first major philosophical doctrine, according to his own confessions. But over the years he distanced himself and, eventually, became a vehement critic of Marxist-Leninist “ontology and politics” [27, 31, p. vii].

3 For example, although Bunge has made a stronger emphasis toward *systems*, his recent writing is still rich in references to *things*, including in the same texts where he discusses systems being preferable to the notion of things [28, p. 174].

4 https://scholar.google.com/citations?user=7MmcYgEAAAAJ&hl=en&oi=ao.

5 Bunge describes systems in [22, p. 270], and in many other sources. To obtain a more detailed discussion of properties of systems, one can consult, for example, [26, pp. 10–19].

6 This was during a personal meeting with Mario Bunge at his residence in Montreal, Canada in September 2018.

7 Bunge [22, p. 270] also distinguishes a *symbolic* (or *semiotic*) system as a type of a concrete system some components of which stand for or represent other objects.

8 This may potentially resolve the criticisms levied against Bunge’s ontology as being too physicalist [76, 121]. The original ideas of Bunge captured in BWW without explicit qualification have indeed been casted by BSO as belonging only to material reality.

9 Here, we see a reference to the CESM model (composition, environment, structure), but the *mechanism* as a component was only developed by Bunge in the early 2000 s, and thus is missing from this passage written in 1985.
